# Heterogeneous Light Supply Affects Growth and Biomass Allocation of the Understory Fern *Diplopterygium glaucum* at High Patch Contrast

**DOI:** 10.1371/journal.pone.0027998

**Published:** 2011-11-23

**Authors:** Wei Guo, Yao-Bin Song, Fei-Hai Yu

**Affiliations:** 1 College of Nature Conservation, Beijing Forestry University, Beijing, China; 2 State Key Laboratory of Vegetation and Environmental Change, Institute of Botany, Chinese Academy of Sciences, Beijing, China; Lakehead University, Canada

## Abstract

Spatial heterogeneity in resource supply is common and responses to heterogeneous resource supply have been extensively documented in clonal angiosperms but not in pteridophytes. To test the hypotheses that clonal integration can modify responses of pteridophytes to heterogeneous resource supply and the integration effect is larger at higher patch contrast, we conducted a field experiment with three homogeneous and two heterogeneous light treatments on the rhizomatous, understory fern *Diplopterygium glaucum* in an evergreen broad-leaved forest in East China. In homogeneous treatments, all *D. glaucum* ramets in 1.5 m×1.5 m units were subjected to 10, 40 and 100% natural light, respectively. In the heterogeneous treatment of low patch contrast, ramets in the central 0.5 m×0.5 m plots of the units were subjected to 40% natural light and their interconnected ramets in the surrounding area of the units to 100%; in the heterogeneous treatment of high patch contrast, ramets in the central plots were subjected to 10% natural light and those in the surrounding area to 100%. In the homogeneous treatments, biomass and number of living ramets in the central plots decreased and number of dead ramets increased with decreasing light supply. At low contrast heterogeneous light supply did not affect performance or biomass allocation of *D. glaucum* in the central plots, but at high contrast it increased lamina biomass and number of living ramets older than annual and modified biomass allocation to lamina and rhizome. Thus, clonal integration can affect responses of understory ferns to heterogeneous light supply and ramets in low light patches can be supported by those in high light. The results also suggest that effects of clonal integration depend on the degree of patch contrast and a significant integration effect may be found only under a relatively high patch contrast.

## Introduction

Light, water and nutrient resources are heterogeneously distributed in natural habitats [1], [2], and horizontal spreading by clonal growth enables interconnected, genetically identical individuals (ramets) of clonal plants to experience patches differing in resource supply [3], [4]. In the past decades, many studies have addressed the effects of heterogeneous resource supply on morphology, growth, reproduction and biomass allocation of clonal angiosperms [5]–[22], but few have examined those effects in clonal pteridophytes. Most pteridophytes are capable of clonal growth and pteridophytes represent an evolutionarily and phylogenetically important group in plant kingdom [23]. Therefore, examining how pteridophytes adapt to environmental heterogeneity will broaden our view on the significance of clonal growth in plants [24].

Light is a limiting factor in forest understory, and individuals of non-clonal plants or isolated ramets of clonal plants commonly respond to reduced light supply by reducing growth, elongating stem/petiole internodes and decreasing root to shoot ratio [5], [12], [13], [20], [22]. In forest understory, light is heterogeneously distributed not only in the vertical layer but also in the horizontal space [25]. Rhizomatous pteridophytes are commonly the dominant species in the understory of many forests and play a key role in forest regeneration [26]–[28]. Studies on the effects of heterogeneous light supply on clonal angiosperms showed that due to clonal integration (i.e. translocation of resources such as carbohydrates, nutrients and water among interconnected ramets of the same clone) performance of ramets growing in low light conditions could be greatly improved when they were connected to ramets growing in high light conditions [5], [9], [12], [14], [29]–[31], and heterogeneous light supply could also change biomass allocation of the ramets [9], [12], [30]. To our knowledge, however, very few studies have examined the effects of heterogeneous light supply on growth and biomass allocation of understory pteridophytes.

Patch contrast, i.e. the degree of the difference in the availability of resources between adjacent patches [32], [33], may greatly affect the pattern of resource translocation between interconnected ramets of clonal plants and thus modify the responses of ramets to local resource supply [33], [34]. In forest understory, there is a gradient of light supply due to the existence of gaps and differences in biological characteristics of tree species (e.g. broad leaf *vs.* coniferous leaf), and understory clonal herbs are likely to grow in patchy environments with different light contrast. A theoretical study has shown that high patch contrast may induce resource translocation between interconnected ramets located in different resource patches and thus affect responses of clonal plants to heterogeneous resource supply, but low contrast may not [34]. However, empirical evidence for the role of patch contrast is still rare [18], [22], [35], [36].

To test the hypothesis that clonal integration can modify the responses of pteridophytes to heterogeneous light supply, we conducted a field experiment with three homogeneous light treatments and two heterogeneous light treatments differing in the degree of patch contrast on the rhizomatous fern *Diplopterygium glaucum* in the understory of an evergreen broad-leaved forest in East China. In the homogeneous treatments, all *D. glaucum* ramets in 1.5 m×1.5 m experimental units were subjected to 10, 40 and 100% natural light, respectively. In the heterogeneous treatment of low patch contrast, *D. glaucum* ramets in the central 0.5 m×0.5 m plots of the experimental units were subjected to 40% natural light, while the ramets in the 0.5-m-wide zone surrounding the central plots (i.e. in the remaining part of the units) were subjected to 100% natural light; in the heterogeneous treatment of high patch contrast, *D. glaucum* ramets in the central plots were subjected to 10% natural light, while the ramets in the surrounding zone to 100%. We expected that (1) in the homogeneous treatments performance (ramet number and biomass) of *D. glaucum* in the central plots would decrease significantly with decreasing light supply; (2) performance of *D. glaucum* in the central plots subjected to 10 and 40% natural light would be larger if the ramets in the surrounding zone were subjected to 100% natural light than to 10 and 40%, i.e. performance of ramets in the central plots would be affected by clonal integration; (3) clonal integration would also affect biomass allocation of the ramets in the central plots subjected to 10 and 40% natural light; (4) the effects of clonal integration would be larger if patch contrast was larger, i.e. the differences in performance of *D. glaucum* in the central plots between the homogeneous and corresponding heterogeneous light treatments would be larger if the central plots were subjected to low light (10% natural light) than to medium light (40% natural light).

## Materials and Methods

### The species

*Diplopterygium glaucum* (Thunb. ex Houtt.) Nakai (Gleicheniaceae), synonymous as *Gleichenia glauca* Hook and *Hicriopteris glauca* (Thunb.) Ching, is an understory fern and propagates via rhizomes that form vertical, perennial fronds (i.e. ramets) with adventitious roots [23], [37], [38]. The diameter of the rhizomes is about 3 mm [38], and the mean distance between adjacent ramets along a rhizome is 8.2 cm [37]. This species is evergreen; new ramets start to come out in April or May and do not wither during winter at normal conditions (Bing-Yang Ding and Teng Fang personal communications). Both rhizomes and aboveground parts of ramets can last at least several years (B–Y. Ding and T. Fang personal communications). In the first year, a stalk with a node, a pair of opposite rachises with pinnae and a bud between the two rachises is produced; this frond with one stalk node corresponds to a current-year ramet and is thereafter called an “annual ramet”. During the second year, the bud produces another internode and node with a new pair of rachises and a new bud, and the process repeats each year (B–Y. Ding personal communications). The ramets that have more than one stalk node and thus are more than one year old are thereafter called “ramets older than annual”. This species mainly inhabits the understory of evergreen forests in mountain ravines at altitudes below 1500 m a.s.l. in east and southeast China [23], [37], [39].

### Study site

The experiment was conducted in the understory of an evergreen broad-leaved forest (29°14′46.9″ N−29°14′50.7″ N, 118°06′59.1″ E−118°07′16.8″ E, 384–553 m a.s.l.) in the Gutianshan National Nature Reserve, located in Kaihua County in Zhejiang Province, China. The study area has a subtropical, moist, monsoon climate, with 1334 hours of sunlight and 250 days of frost-free period [39]. The mean annual precipitation is 1964 mm and mean temperature is 15.3 °C [40]. In this old-growth forest *D. glaucum*, with around 20% coverage, is the most dominant species in the herb layer [26].

### Experimental design

**Figure 1:**
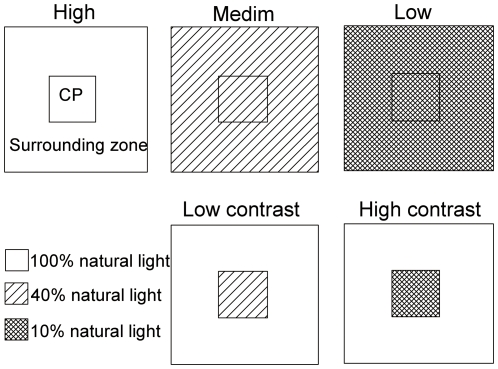
Experimental design. Each experimental unit (1.5 m×1.5 m) consists of a 0.5 m×0.5 m central plot (CP) surrounded by a 0.5-m-wide zone (surrounding zone). High, Medium and Low stand for homogenous, high, medium and low light supply, i.e. the whole unit received 100%, 40% and 10% natural light, respectively; Low contrast and High contrast represent heterogeneous, medium and low light supply, i.e. the central plot of the unit received 40% and 10% natural light, respectively, and the surrounding zone received 100% natural light.

We set up ten blocks in the understory dominated by *D. glaucum*. Each block was 15 m×15 m in size and consisted of five experimental units of 1.5 m×1.5 m (Fig. 1). There were 20–30 ramets of *D. glaucum* in each experiment unit and the distance between any of the two adjacent units was at least 2–4 m. Each unit consisted of a central plot of 0.5 m×0.5 m with 6–9 ramets and a 0.5-m-wide zone surrounding the plot (Fig. 1). The five units in each block were randomly subjected to one of the five light treatments: (i) homogeneous, high light supply (coded as “High”), i.e. the whole unit received 100% natural light in the forest understory; (ii) homogeneous, medium light supply (code as “Medium”), i.e. the whole unit was covered with one layer of black shading net and received 40% natural light; (iii) heterogeneous, medium light supply with a relatively low degree of patch contrast (code as “Low contrast”), i.e. the central plot of the unit received 40% natural light and the surrounding zone received 100%; (iv) homogeneous, low light supply (coded as “Low”), i.e. the whole unit was covered with two layers of black shading net and received 10% natural light; (v) heterogeneous, low light supply with a relatively high degree of patch contrast (coded as “High contrast”), i.e. the central plot received 10% natural light and the surrounding zone received 100%. For shading, black shading net that does not change the ratio of red to far-red light was used to enclose the whole units (for treatments of Medium and Low) or the whole central plots (for treatments of Low contrast and High contrast). The shading nets were hold by bamboo sticks about 1.2 m above the soil level on the top of the units or plots and 0.4 cm above the soil level at the vertical edge.

### Measurements

The experiment started on 3 April 2009 and ended on 1 October 2009. At the beginning of the experiment, we counted number of *D. glaucum* ramets in the central plot and the surrounding zone of each unit, and measured stalk length (height) of each ramet in the central plot. ANOVA showed that number of ramets of *D. glaucum* in the central plots was on average 6.9 (SE = 0.1) and did not differ among the five light treatments (*F*_4,36_ = 1.56, *P* = 0.207) or blocks (*F*_9,36_ = 1.56, *P* = 0.166); mean ramet height in the central plots was on average 58.9 cm (SE = 1.6) and did not differ among the treatments (*F*_4, 36_ = 0.59, *P* = 0.673) or blocks (*F*_9,36_ = 1.69, *P* = 0.128).

After 181 days on 1–3 October, we counted in each central plot number of living *D. glaucum* ramets with one stalk node (i.e. annual ramets), number of living ramets with more than one stalk nodes (i.e. ramets older than annual), and total number of dead ramets. We then harvested all the living plants of *D. glaucum* in the central plots. Plant materials in each plot were separated into lamina, stalk, rhizome and root, and the dry mass was measured after drying at 80°C to constant weight.

### Data analysis

We used ANOVA with a randomized block design to test the effects of light treatment and block on biomass (root, rhizome, stalk and lamina mass and total biomass), proportional biomass allocation and ramet number (number of living, annual ramets, number of living ramets older than annual, total number of living ramets or dead ramets) of *D. glaucum* in the central plots. In these analyses, light was treated as a fixed factor and block as a random one. When a significant treatment effect was detected, Duncan's tests were conducted to compare the differences in trait means among the five light treatments. SPSS 17.0 software (SPSS, Chicago, IL, USA) was used for all analyses.

## Results

There was a significant block effect on final biomass and proportional biomass allocation to root (Table 1B), but not on ramet number (Table 2) or biomass allocation to rhizome, stalk or lamina (Table 1B).

**Table 1:** ANOVA results of effects of light treatments and block on (A) biomass of and (B) proportional biomass allocation to root, rhizome, stalk and lamina of *Diplopterygium glaucum* in the central plots.

		Root		Rhizome		Stalk		Lamina^1^			
Effect	DF	*F*	*P*	*F*	*P*	*F*	*P*	*F*	*P*		
(A) Biomass											
Treatment	4, 36	1.51	0.220	1.39	0.257	5.31	0.002	2.67	0.017		
Block	9, 36	2.93	0.010	2.40	0.030	3.53	0.003	10.56	<0.001		
(B) Proportional biomass allocation											
Treatment	4, 36	0.82	0.521	7.33	<0.001	1.45	0.239	10.74	<0.001		
Block	9, 36	3.29	0.005	1.08	0.403	1.33	0.256	0.75	0.666		

1 Data on lamina biomass were ln-transformed before analysis.

**Table 2:** ANOVA results of effects of light treatments and block on ramet number of *Diplopterygium glaucum* in the central plots.

		Total no. of living ramets		No. of living annual ramets		No. of living ramets older than annual		Total no. of dead ramets	
Effect	DF	*F*	*P*	*F*	*P*	*F*	*P*	*F*	*P*
Treatment	4, 36	6.31	0.001	0.88	0.488	9.53	<0.001	7.74	<0.001
Block	9, 36	1.42	0.217	1.27	0.284	0.78	0.640	1.37	0.236

### Effects of light supply on performance traits

**Figure 2:**
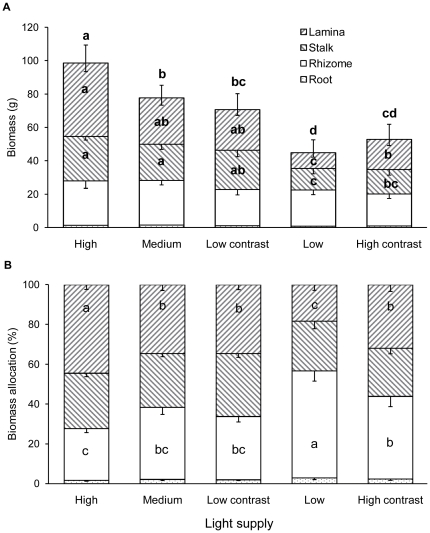
Biomass and biomass allocation of *Diplopterygium glaucum* in the central plots under the five light treatments. Treatment codes are as in Fig. 1. Bars and vertical lines represent mean and SE. Bars sharing the same letters are not different at *P* = 0.05. Letters above the bars are for the test for total biomass, and those inside bars are for the tests for each plant part.

**Figure 3:**
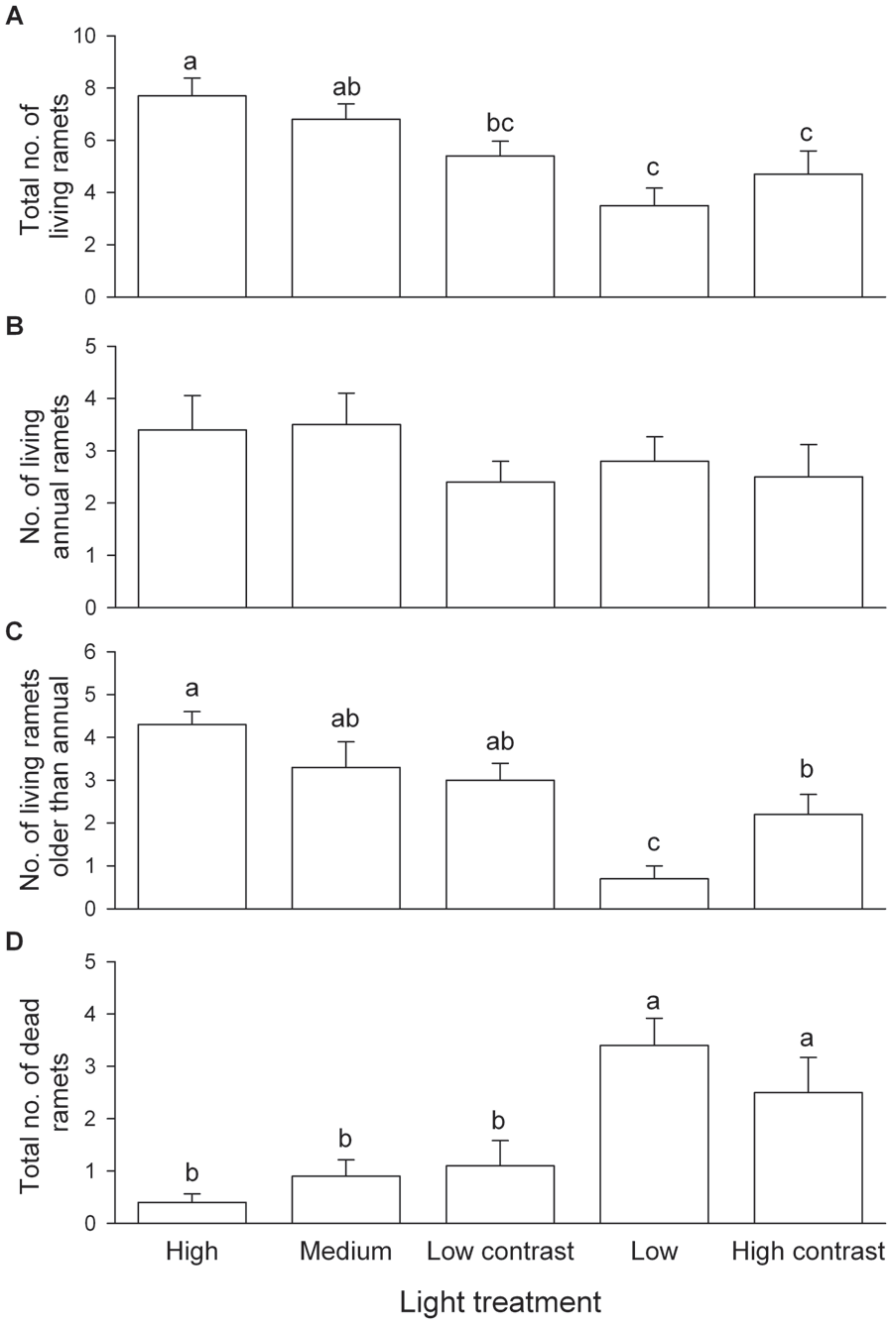
Number of ramets of *Diplopterygium glaucum* in the central plots under the five light treatments. (A) Total number of living ramets, (B) number of living, annual ramets, (C) number of living ramets older than annual and (D) total number of dead ramets. Treatment codes are as in Fig. 1. Bars and vertical lines represent mean and SE. Bars sharing the same letters are not different at *P* = 0.05.

The light treatments significantly affected total biomass, stalk biomass, lamina biomass, total number of living ramets and number of living ramets older than annual in the central plots (Tables 1A and 2). In the homogeneous treatments, total biomass, lamina biomass, stalk biomass (Fig. 2A), total number of living ramets (Fig. 3A) and number of living ramets older than annual (Fig. 3C) in the central plots decreased significantly with decreasing light supply, but total number of dead ramets increased (Fig. 3D).

Despite the degree of patch contrast, heterogeneous light supply did not significantly affect total biomass, stalk biomass, rhizome biomass, root biomass (Fig. 2A), total number of living ramets or total number of dead ramets (Fig. 3A and D) in the central plots (Low contrast *vs.* Medium and High contrast *vs.* Low). At low patch contrast heterogeneous light supply also did not affect lamina biomass or number of ramets older than annual (Low contrast *vs.* Medium), but at high patch contrast these two variables were significantly larger in the heterogeneous than in the homogeneous treatment (High contrast *vs.* Low; Figs. 2A and 3C).

### Effects of light supply on biomass allocation

The light treatments significantly affected proportional biomass allocation to rhizome and to lamina in the central plots, but not that to stalk or root (Table 1B). In the homogeneous treatments, biomass allocation to lamina decreased significantly with decreasing light supply, whereas that to rhizome increased (Fig. 2B).

At low patch contrast heterogeneous light supply did not affect proportional biomass allocation to lamina or rhizome in the central plots (Low contrast *vs.* Medium), but at high patch contrast biomass allocation to rhizome was significantly smaller and that to lamina larger in the heterogeneous than in the homogeneous treatment (High contrast *vs.* Low; Fig. 2B).

## Discussion

In the homogeneous treatments, decreasing light supply greatly reduced performance (biomass and ramet production) of *D. glaucum* in the central plots, suggesting that light is a factor limiting the growth and distribution of *D. glaucum* in the understory of the evergreen forest. The results also suggest that the two degrees of patch contrast that we set up in the heterogeneous treatments may potentially induce translocation of resources (most likely carbohydrates) because the growth of ramets growing in the different light patches differed greatly. However, we might underestimate the effects of shading because there might exist relative long-distance clonal integration, i.e. shaded ramets in the central plots could receive carbohydrate support from unshaded ramets outside the whole units.

The *D. glaucum* ramets in the central plots subjected to 40% natural light did not produce more biomass or more living ramets in the heterogeneous than in the homogeneous medium light treatment. This was very likely because at low patch contrast either there was no translocation of resources from the unshaded ramets in the surrounding zone to the shaded ramets in the central plots or the translocation was insufficient to affect their performance. However, the *D. glaucum* ramets subjected to 10% natural light produced more lamina biomass and more living ramets older than annual in the heterogeneous than in the homogeneous low light treatment, indicating that at high patch contrast there was significant resource translocation and clonal integration increased performance of the shaded ramets. This result agrees with previous findings that preventing resource translocation by severing rhizome connection significantly reduced survival and growth of individual ramets of the same species [37] as well as two lycopods, *Lycopodium flabelliforme* [40] and *Diphasiastrum digitatum* [24]. The results also suggest that patch contrast is a key factor that influences the patterns and effects of resource translocation in clonal ferns, agreeing with the findings on clonal angiosperms that increasing patch contrast can increase resource translocation [22], [35].

One may argue that we did not find a significant effect between the homogeneous and heterogeneous, medium light treatment might be because there existed relative long-distance clonal integration between shaded ramets in the central plots and unshaded ramets outside the experimental units. The effects of this long-distance integration were strong enough to cover the effects of the short-distance clonal integration between shaded ramets in the central plots and unshaded ramets surrounding the plots within the units. However, we did find significant differences between the homogeneous and the heterogeneous low light treatment. This suggests that, even if there was long-distance integration, its effects were not as strong as those of the short-distance integration.

Even at high patch contrast, theterogeneous light supply did not affect rhizome or root biomass of *D. glaucum* in the central plots and thus did not influence total biomass. Also, in the homogeneous treatments decreasing light supply did not change rhizome or root biomass. The likely reason for this irresponsiveness to light supply is that large amount of rhizome and root biomass of the perennial fern *D. glaucum* was gradually accumulated during many years, and the duration of the experiment (about six months) may not be long enough to affect the underground biomass of the species [23]. As a result, clonal integration did not affect total biomass of the ramets in the central plots, despite an integration-mediated increase in lamina biomass.

Heterogeneous light supply significantly affected proportional biomass allocation of *D. glaucum* in the central plots at high patch contrast (i.e. the ramets in the central plots were subjected to low light) but not at low patch contrast (i.e. the ramets in the central plots were subjected to medium light), suggesting again that increasing patch contrast can significantly increase the amount and thus the effect of resource translocation [18], [22], [34], [35], [36]. At high patch contrast, heterogeneous light supply significantly increased biomass allocation to lamina and decreased that to rhizome. This was because at high patch contrast clonal integration markedly increased lamina biomass, slightly decreased rhizome biomass, and did not affect root or stalk biomass of *D. glaucum* in the central plots. Thus, clonal integration enabled *D. glaucum* to enhance not only the absolute amount of light harvest by increasing lamina biomass (and thus lamina area) but also the relative amount by increasing ratio of above- to belowground resource absorption. These integration-mediated responses may have helped the understory fern *D. glaucum* to tolerate some extremely low light conditions and contributed greatly to the success of the populations in forest understory where light is highly heterogeneously distributed.

This study provides evidence that heterogeneous resource supply can affect performance and biomass allocation of clonal pteridophytes. Our results also suggest that the effects of resource translocation (clonal integration) induced by heterogeneous light supply on clonal pteridophytes may depend on the degree of patch contrast and a significant effect of clonal integration may be found only at a relatively high patch contrast.
